# Forearm compartment pressures and grip strength in elite motorbike racers with chronic exertional compartment syndrome

**DOI:** 10.1186/s13018-021-02765-z

**Published:** 2021-10-15

**Authors:** Dominic P. O’Dowd, Heike Romer, Richard Hughes, Noel Harding, Samantha Ball, Filippo Migliorini, Nicola Maffulli

**Affiliations:** 1 Centre for Sports and Exercise Medicine, Barts and The London School of Medicine and Dentistry, Mile End Hospital, 275 Bancroft Road, London, E1 4DG England; 2 Medical Team, British Superbike Series, MSVR Brands Hatch, West Kingsdown, Longfield, DA3 8NG UK; 3 Department of Anaesthesia, Royal Liverpool and Broadgreen University Hospital NHS Trust, Liverpool, UK; 4 Department of Orthopaedic, Trauma, and Reconstructive Surgery, RWTH University Hospital, Pauwelsstraße 30, 52074 Aachen, Germany; 5 Department of Musculoskeletal Disorders, Faculty of Medicine and Surgery, University of Salerno, 84081 Baronissi, Salerno, Italy; 6 School of Pharmacy and Bioengineering, Keele University School of Medicine, Stoke on Trent, UK

**Keywords:** Chronic exertional compartment syndrome, Compartment pressure, Forearm, Upper limb, Grip strength

## Abstract

**Background:**

Remarkably little research has been published on chronic exertional compartment syndrome (CECS) of the forearm. This study investigated forearm flexor compartment pressure pre- and post-exercise in elite motorbike racers clinically diagnosed with CECS and assessed their grip strength before and after arm pump exercise.

**Methods:**

Elite motorbike riders with a clinical diagnosis of CECS of the right forearm when racing were recruited during the opening rounds of a British Superbike season. Their grip strength and forearm flexor compartment pressures were measured before and after a set exercise programme.

**Results:**

Of the 11 riders recruited to the study, 10 completed the full testing regime. The mean pre-exercise forearm compartment pressures [11.7 mmHg (range 7–17 mmHg)] significantly increased post-exercise [30.5 mmHg (range 15–45 mmHg)], with a mean increase of 18.80 mmHg (*P* < 0.0001). The mean pre-exercise grip strength [50.61 mmHg (range 37–66.7 mmHg)] decreased post-exercise to [35.62 mmHg (range 17.1–52.5 mmHg)], a mean decrease of 14.99 mmHg (*P* < 0.0001).

**Conclusion:**

There is a statistically significant increase in the forearm flexor compartment pressures in elite motorbike racers with CECS, but with marked variability of these values. Grip strength decreases statistically significantly following onset of symptoms of CECS of the forearm.

## Introduction

Chronic exertional compartment syndrome (CECS) was first described in 1956 in the anterior tibial compartment in a footballer [1]. This condition was not recognised as exercised-induced compartment syndrome in the upper limb until 1984, and it is still less well documented compared to CECS in the lower limb [2–4]. In the upper limb, CECS typically presents with exercise-induced pain and tightness of the forearm, with occasional paraesthesiae and loss of forearm muscle control. The symptoms resolve rapidly after cessation of exercise, only to recur with further exertion [3, 4]. Although reported in manual workers, the condition is most common in athletes who require sustained and excessive use of their upper limbs to perform in their particular sport [5–9]. Baseball pitchers, body builders, cyclists, kayakers, motorcyclists, rock climbers, rowers, swimmers, water skiers, wheelchair athletes and wind surfers have all been reported to suffer from CECS of the upper limb [9–15]. CECS is common in motorcyclists, in whom typically the flexor compartments of the right forearm are affected, likely from a combination of gripping onto the bike during rapid acceleration/deceleration and prolonged and/or excessive force applied on the brake lever. Riders typically report that braking becomes progressively more difficult and that braking worsens symptoms, potentially endangering the safety of the affected rider. CECS is a diagnosis of clinical suspicion, validated by the measurement of high pressure within the compartment following exercise and possibly at rest [16]. Pedowitz’s modified criteria to diagnose CECS were originally defined from lower limb pressure studies and have subsequently been modified and adopted by some authors to diagnose upper limb CECS [3]. However, the pressure thresholds used to diagnosis forearm CECS are still debated [17]. A handful of papers suggest varying diagnostic values for resting and post-exercise forearm pressures as well as differing times for measurement post-exercise in symptomatic individuals [6, 16, 18, 19]. Two studies have reported normative forearm compartment pressures in asymptomatic individuals [20, 21]. We are unaware of any study which has specifically focused on patients with forearm CECS and their correlating grip strength.

This study aimed to determine the difference in pre- and post-exercise forearm compartment pressures and the effect on grip strength in elite motorcycle racers with a clinical diagnosis of CECS of the right forearm.

## Methods

All procedures described in the present investigations were approved by the Research and Ethics Committee of Queen Mary University of London. The British Superbikes medical team was also made aware of and involved with the study (Table 1).

**Table 1:** Right forearm flexor compartment pressures

Rider ID	Pre-exercise pressure (mmHg)	Post-exercise pressure (mmHg)	Pressure change (mmHg)	Pressure change (%)
01	7	15	8	214
02	16	34	18	213
03	7	25	18	357
04	8	45	37	563
05	16	32	16	200
06	17	32	15	188
07	7	27	20	386
08	13	32	19	246
09	15	34	19	227
10	11	29	18	264
11	28	Rider withdrew from study		
Overall	11.7	30.5	18.8	261

Male motorcycle riders aged between the 16 and 65 with right forearm pain suggestive of CECS who were competing in any class at the British Superbike (BSB) championship were eligible to take part in the study. They were recruited via information leaflets displayed around the paddock and referral from those attending the series physiotherapy services. Exclusion criteria were previous fasciotomy of the forearm, minor forearm injury in the last 2 weeks, major forearm injury in the last 6 months, symptoms suggestive of a different pathology. We also excluded subjects taking anticoagulants, allergic to local anaesthetic, or needle phobic. Following a clinical diagnosis of CECS, verbal recruitment and information sharing, informed written consent was obtained from each rider. Subjects then filled in a study questionnaire about their symptoms, a pre- and post-exertion Visual Analogue Scale (VAS) pain score, previous treatments and the racing class that they competed in (Table 2).

**Table 2:** Right hand grip strength

Rider ID	Pre-exercise strength (mmHg)	Post-exercise strength (mmHg)	Strength change (mmHg (%))
01	55.8	41	− 14.8 (− 26.5)
02	37.0	17.1	− 19.9 (− 53.8)
03	48.5	39.4	− 9.1 (− 18.8)
04	44.7	36.4	− 8.3 (− 18.6)
05	49.3	35.5	− 13.8 (− 28)
06	40.3	28.2	− 12.1 (− 30)
07	63.5	38.0	− 25.5 (− 40.2)
08	66.7	52.5	− 14.2 (− 21.3)
09	45.1	32.2	− 12.9 (− 28.6)
10	55.2	35.9	− 19.3 (− 35)
11	50.3	Rider withdrew from study	

**Fig. 1:**
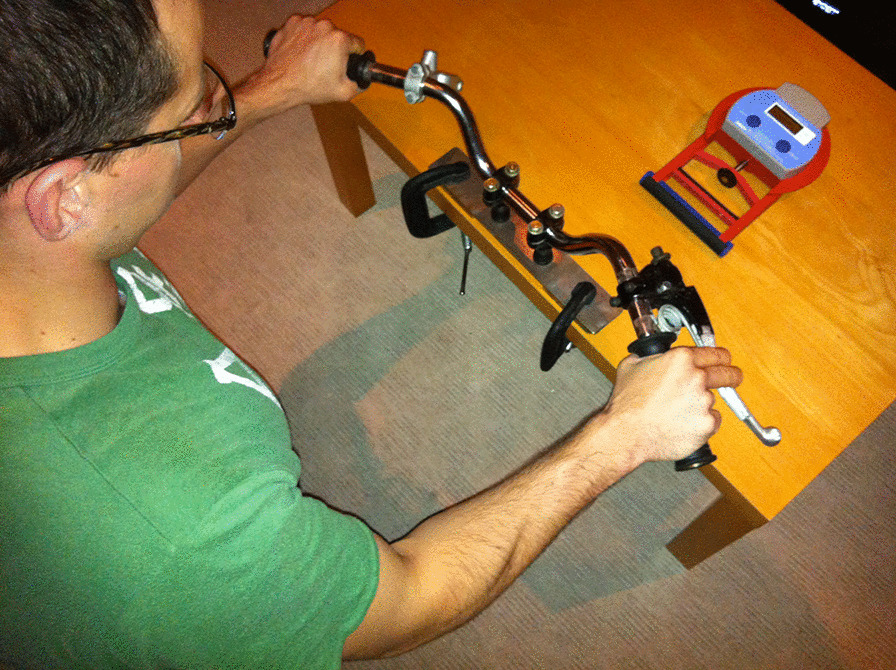
Static motorbike handlebars set up for exercise testing

Riders sat behind a mock up set of motorcycle handlebars with a brake lever fixed by a metal spring. Local anaesthetic 0.5% Bupivacaine (Middlesex, UK) 3–5 ml was injected into the skin at the junction of the proximal and middle third of the antero-medial aspect of the forearm. Using a calibrated portable Stryker® (Kalamazoo, MI, US) Intracompartmental Pressure Monitor System, a side-ported needle was inserted in a proximal to distal, radial to ulnar direction, at 45° to horizontal, through the skin into the deep flexor compartment of the right forearm. All measurements were obtained with the hand supinated and the elbow extended. Resting forearm compartment pressures were obtained, and the needle was removed. Following removal of the needle, resting grip strength in the right arm was immediately measured using a Takei® (Yashiroda, Niigata, Japan) T.K.K 5401 Grip D handheld grip strength dynamometer, which has been validated as accurate and reliable apparatus for repeated grip strength measurements [22]. There is no agreed standard exercise testing programme to reproduce symptoms of forearm CECS. Using the handle bars clamped to a table, subjects were asked to grip the brake lever as hard as they could and as many times as they could over a 2 min period, to replicate braking during a race and reproduce forearm symptoms. This exercise was devised to replicate racing activity following discussion with riders, experienced physiotherapists and a consultant specialising in forearm CECS in motorcyclists. The brake lever had a spring of fixed strength attached to the handlebars (in place of the usual hydraulic system) to provide comparable resistance to normal race breaking when the brake lever was gripped. The spring was calibrated by a non-clinical member of the research team to replicate 150PSI of pressure, as breaking maximum is usually 150PSI/10Bar before locking up the front end on a dual disc bike (Fig. 1).

Immediately following cessation of the standardised exercise challenge, post-exercise intra-compartmental pressure was taken. The portable Stryker® Intracompartmental Pressure Monitor System with side ported needle was recalibrated and reinserted at 45° through the skin into the deep flexor compartment of the right arm at the same insertion site. Following removal of the needle, post-exercise grip strength was obtained using the same machine and method as outlined above. To ensure reliability and continuity, a single investigator supervised the tests and took and recorded all measurements from all subjects in the study.

Data were analysed using SPSS®. Paired *t* tests were carried out on both the pre- and post-exercise compartment pressures and grip strengths.

## Results

A total of 11 (age range 17–29 years, median 21 years) male elite motorcycle riders from the BSB paddock with a clinical diagnosis of CECS took part in this study. They were spread across all the different bike classes except the 125 cc class (which has a large number of riders aged less than 18). The mean duration of symptoms was 33.5 months (range 6–89 months), and the mean VAS symptomatic pain score was 61.6 (range 13–100). All riders stated that their forearm condition affected both their grip strength and their racing performance. Prior to this study, 5 riders had received physiotherapy, and 1 rider had been referred to hospital by his GP for the condition and undergone EMG studies, which were normal. Nine of the 11 riders reported that tracks which have a high average speed, variable track elevation and frequent areas of rapid acceleration and deceleration cause forearm symptoms more often than other tracks without such characteristics. Riders used a VAS to assess whether the symptoms elicited by the exercise they were asked to undertake during the study replicated the symptoms they normal suffer from. Results from this showed a mean of 80 points (range 44–99).

The mean pre-exercise right forearm compartment pressure was 11.7 mmHg (range 7–28 mmHg), increasing post-exercise to a mean 30.5 mmHg (range 15–45 mmHg), a mean change of 18.8 mmHg (range 8–37 mmHg; *P* < 0.0001). Subject 011, who did not complete the test, was not included in the data analysis. His pre-exercise compartment pressure was much greater than that of all the other subjects, and he withdrew from the study.

The mean pre-exercise right hand grip strength of 50.61 mmHg (range 37–66.7 mmHg) decreased post-exercise to a mean of 35.62 mmHg (range 17.1–52.5 mmHg), a mean change in pressure of 14.99 mmHg (range 8.3–25.5 mmHg; *P* < 0.0001). When expressed as a percentage, a mean decrease in grip strength of 30% (range 18.6–53.8%) took place. There was no evidence of a statistically significant association between changes in compartment pressure and changes in grip strength. Equally, there was no evidence of a statistically significant association between the amount of change in compartment pressure and the amount change in grip strength.

## Discussion

This study shows a statistically significant (*P* < 0.0001) increase in forearm compartment pressures in elite motorbike riders with forearm CECS following an exercise challenge, and a consistent and statistically significant (*P* < 0.0001) reduction in grip strength in the affected forearm.

The Pedowitz modified criteria for diagnosis of CECS were developed from lower limb CECS patients [23]. A pressure indicative of CECS is defined as a resting pressure ≥ 15 mmHg and a post-exercise pressure ≥ 30 mmHg or ≥ 20 mmHg 5 min post-exercise. The mean pre-exercise compartment pressures in this present study would not lead to a diagnosis of CECS based upon these guidelines, but the mean post-exercise pressures would meet them. Examining individual subjects, 4 of the 10 riders who fully completed the testing fulfilled both the pre- and post-exercise criteria for diagnosis of CECS, with a further 2 exceeding the post-exercise pressure only. Four of the 10 subjects do not meet the Pedowitz criteria for diagnosis of CECS based on their pre- and post-exercise pressures. This suggests that the Pedowitz criteria may not be fully applicable to CECS of the upper limb or indeed conclusive for diagnosis of CECS at any location. In climbers, Schoeffl et al. concluded that lower leg CECS guidelines cannot be used for the upper limb [19]. All climbers experience ‘pumped’ arms, and therefore, they suggested that CECS should only be diagnosed if forearm pressure was greater than 30 mmHg 15 min after stress. Further forearm CECS research published more recently, with the largest numbers to date, has refined this suggesting a sustained increase in pressure at 14.5 min post-exercise is a more sensitive and specific indicator than peak values alone [18].

Comparing our subjects to those of a study examining pre-exercise pressures in asymptomatic subjects, all the subjects in the present study were within the normal reference range [20]. We used the same type of compartment measuring device used in that study. The reason for this may be the high variability in resting values in Ardolino’s paper or may have resulted from the possible different location on the forearm of measurements between the two studies [20]. Indeed, small changes in measurement location exert significant effects on the pressures measured [21]. The Stryker® Intracompartmental Pressure Monitor System and side-ported needle were chosen as they have both been shown to be valid and reliable tools to measure compartment pressures [24].

The published literature regarding upper limb CECS reports the use of a variety of pressure devices, exercises to reproduce symptoms, and time of pressure measurement in relation to the exercise challenge [5, 9, 19, 25]. Combined with small numbers and retrospective and uncontrolled data collection, this makes comparison inaccurate. We are aware that the present investigation should be considered preliminary, and that a much larger study would be required to provide normative data. Multiple post-exercise pressure measurements were not performed as part of this study, but serial assessment of intra-compartmental pressure should be considered in future research.

The limited sample size and the lack of a control group represent the most important limitations. The measurements were performed at different race venues, but the researcher used the same equipment which was calibrated before and after each test. We are aware that we could have used continuous pressure monitoring, but the use of the slit catheter and tubing was not practical in our setting. This implies that the change in pressure between cessation of exercise and insertion of the monitoring device was not recorded, and that peak pressure during exercise was not recorded. However, this may well imply that the pressures recorded are lower than the actual pressure reached during exercise. Development of reliable pressure monitoring equipment for field use is required to tackle this limitation. Given the restrictive nature of the protective leather suit riders wear, testing had to be artificially reproduced rather than undertaken immediately after riding given the likelihood that pressures would have normalised by the time of measurement. All riders completed a VAS scoring chart, which showed symptoms during testing correlated well with symptoms experienced during riding, with a mean score of 80/100. The difference between the subjective symptom scoring related to actual racing and those in the study conditions undoubtedly represents the complex multifaceted forces that occur during racing. A further limitation is that we were not able to reproduce the G forces of acceleration and deceleration, the forces of braking and throttle twist acceleration, and the fine control of balance and steering required in constantly altering positions whilst on the bike. We are aware that intra-compartmental pressures and grip strength may therefore be different when additional movement and G forces are present. The exact effect that this would have on pressures and grip strength is unclear.

## Conclusion

A simulated motorbike riding exercise increases upper limb compartment pressures in subjects with clinical symptoms of CECS and also confirms the variability of pressure measurements in this condition. This is the first study we are aware of which demonstrates that grip strength significantly decreases when symptoms of CECS of the upper limb are present. Further research with larger numbers of subjects and continuous pressure monitoring needs to be undertaken to establish a more reliable reference range for pre-, peak and post-exercise pressures in subjects with symptoms of forearm CECS. Studies aiming to prevent CECS in motorbike riders may ultimately lead to improved motorbike control for amateur and professional riders when racing, which could potentially improve both performance and safety.

## Data Availability

This study does not contain any third material.

## Abbreviations

VAS: Visual Analogue Scale
BSB: British Superbike
CECS: Chronic exertional compartment syndrome
